# Medicine in Paris

**Published:** 1841-09

**Authors:** 


### MEDICINE IN PARIS.

*London, May* 29, 1841.

* * * * * "As respects the matters of our own profession, and the men most prominently concerned in them, I shall unite them under the same head, and offer a few remarks on them in that capacity. Of these things, in the two great capitals, London and Paris, which embody the best and the worst of all places on earth, I have not yet seen as much (so deeply have I been engrossed in business) as I purpose to see hereafter—nor have I perhaps seen enough to justify me in speaking positively of them, in all their characteristics and bearings. But I have seen a sufficiency of them, especially in Paris, to authorize me to assert of them unhesitatingly, and in the most unqualified manner, that, like most other things, they (in common parlance) '*loom largest at a distance.*' As you approach and scrutinize them, they sustain, in their magnitude and importance a strong-marked diminution.

"In Paris (I speak of the Hospitals, attended of course by the most eminent men the capital contains) true *disease-curing* and *life-preserving* medicine and surgery are but little known or regarded—most assuredly they are but little *practised.* Into those ancient and vast repositories of disease and misery, whose walls are taught to echo but to sighs and moans, patients are received but to be *ausculted* and otherwise *diagnostically* treated, die and be *post-mortemized*, for the instruction of the *eleves, internes* and *externes*, and the improvement of the profession by furnishing the *material* for writers and printers—and there the process ends. As it has appeared to me, an anxiety to

*cure* the sick and suffering, entrusted to their care, neither disturbs the repose, nor interferes in any way or degree, with the jollity and enjoyments of the hospital practitioners, whatever may be the departments of the profession in which they labor. If, by post-mortem inspection, the *physician* can prove the accuracy of his diagnosis, his end is attained. And the surgeon is satisfied with a dextrous and showy operation, though the subject of it should die, and be under the dissecting knife of some hawk-eyed *hospital-walker*, in an hour afterwards. Nor are these facts, extraordinary and even *fictitious* as they may appear, either denied by the Faculty of Paris, or complained of by the public. It was distinctly stated to me, by a gentleman, whose information, accuracy and regard for truth I could not question, that he had heard M. V. and other surgeons openly say, when preparing to operate, 'this patient will certainly die, perhaps the *sooner* for the operation; but the profession *may* be benefited by it. We shall therefore proceed.' Accordingly, with finished tact and adroitness, and, with the utmost degree of show of every thing but *feeling*, the instruments are flourished, and the operation performed—and the patient (all at least that remains of him) is promptly removed, that the table may be ready for the reception of another, who is similarly dealt with—and thus—and thus—until the drama of the morning is closed. I say 'of the morning,' and very early in the morning too; for the physicians and surgeons never visit the hospitals at any other time. And, for *surgery*, the custom is attended with no *great* evil—if with *any at all*. But, for the *practice of medicine*, it is fraught with mischief—the morning, as every one knows, being the time when the most characteristic and instructive symptoms of disease are exhibited in the faintest and least *communicative form and degree*. True; auscultation and the other elements of diagnosis and symptom-hunting may be then practised with perhaps sufficient accuracy; and they make nearly the whole subject of the practitioner's regard. I ought to have said, of the *expectant's* regard. For the symptoms and diagnoses being mastered, and announced, the physician considers his duty performed—at least he might as well so consider it; for in the form of actual practice and cure, he does positively nothing. The plan he pursues, with his *three*, *four*, or *five* leeches, applied to the body of an adult, his gum and sugar-water potions, his thimble-full or two of magnesia, his lavements of tepid water, and his other foolery of the like description—such professional trumpery as this, constitutes as literally the

*medicine expectante,* as even the imposture of Hahnemann and his followers, when they administer their drugs in *millionths* of grain doses! Nor is it by the *medical* attendant alone that this farce is enacted. The course of the *surgeon* is nearly the same. The *operation* being performed, his patient is surrendered to the manipulations of others; while he perhaps never sees him or thinks of him again.

From these considerations, and many other kindred ones, which cannot be embodied in the compass of a letter, I have no hesitation in believing that Parisian practice, both medical and surgical, is more successless than that pursued in any other *cultivated* portion of the globe. And indubitably more so than that in any *uncultivated* one; where, in cases of disease, nature, in her undiminished vigor, and in a course free at once from restraint and obstruction, is left almost entirely to her own resources, in her efforts to restore health. In verification of these remarks, take the following facts, extracted from innumerable others, which might be cited.

"The Hotel Dieu is at once the oldest, I believe the largest, and certainly the most celebrated hospital in the world. And its attending Faculty rank with the ablest that Europe can boast. Yet, when the *cholera* came down on Paris, in 1830 or 31, of the first 600 patients received into that *great mortuary,* only ONE survived; and of the first 1000 only FIVE!!—a spectacle of mortality never witnessed *before*—nor even *then,* in any other portion of the world.

"For this surpassing degree of mortality in Paris, there no doubt exist other causes, apart from the unskilfulness of physicians and surgeons. Inattention and recklessness do their part in the dismal waste of life. Notwithstanding their urbanity, kindness, keen sensibilities, and vivid sympathies, the French set, in the abstract, a lower estimation on life, and are therefore more regardless of saving it, than any other people I have ever known—and I believe of any that exist. They realize, in its full extent, the great truth, that '*man is born to die;*' and of the time and manner of death, they make but little account. And this sentiment, if I mistake not, makes its way into the practice of medicine and surgery. Hence the struggle of the practitioner to triumph over disease is neither eager nor obstinate. Nor is this all.

"The mass of Parisian hospital patients are of the *lowest orders* of the *low;* whose subsistence and mode of life are inconceivably wretched. Hence the stamina of their systems, originally feeble and defective, are rendered much more so, by subsequent causes, such as scanty and unwholesome food, a vitiated atmosphere, labor beyond what they can bear with impunity, and, in too many instances, life-wasting dissipation and profligacy. No wonder, therefore, that when under the shattering influence of so many deleterious and powerful agents, they are unable to sustain the shock of disease. I need hardly add, that the causes just cited, which modify so deeply and injuriously the human constitution, must necessarily inflict on it complaints correspondingly modified in form and malignity.

"Under all these circumstances, which are notoriously true, is it not matter of astonishment, that, in the United States, the Parisian schools of medicine and surgery are held in such supreme estimation, as we know is attached to them? Admit, if you please, that French practice *may* cure *French men;* it *kills* Americans, as numberless facts bear positive testimony. The following is one of them:

"A young American, who, as the phrase runs, was walking the French hospitals, was attacked last autumn by *enteritis*, and placde himself under the care of M. V., one of the most celebrated of the Parisian Faculty, both as a teacher and a practitioner. For this disease, which was very violent of its kind, the prescription was *five* leeches to the abdomen, water sweetened with beet-sugar to be taken *ad libitum*, and a *lavement* of cold water to be administered *twice* a day. Meantime another young American, then in Paris, who had practised medicine about a year in South Carolina, visited his sick friend, and, deeming his complaint seriously threatening, entreated him to take immediately an active purgative, composed chiefly of calomel and opium, to have a vein opened, and a large blister applied to the abdomen. In deference to the French physician, the advice was neglected; and in forty-eight hours more, the patient was a corpse!

"Still however, it is regarded as an important feather in the cap of a young physician, in the United States, to have passed some time, and expended his money, in attendance on schools and *amusements* in Paris, and in strolling through Switzerland, and visiting Rome, and other parts of Italy. On this fashionable tour of education, I shall perhaps make known my opinion more fully hereafter. I shall only, at present, therefore, express my conviction, that, in most cases, it is not only *useless*, but *injurious*—and too often productive of absolute ruin." C. C.

